# Concept analysis of conscience-based nursing care: a hybrid approach of Schwartz-Barcott and Kim’s hybrid model

**DOI:** 10.1186/s12910-024-01070-8

**Published:** 2024-06-18

**Authors:** Soheyla Kalantari, Mahnaz Modanloo, Abbas Ebadi, Homeira Khoddam

**Affiliations:** 1 Nursing Research Center, Golestan University of Medical Sciences, Gorgan, Iran; 2 Research Center for Life & Health Sciences & Biotechnology of the Police, Direction of Health, Rescue & Treatment , Police Headquarter, Tehran, Iran

**Keywords:** Nursing, Conscience, Ethics, Nursing, Decision making, Patient care, Professionalism, Thematic analysis, Qualitative research, Competence, Spirituality

## Abstract

**Background:**

The nursing profession considers conscience as the foundation and cornerstone of clinical practice, which significantly influences professional decision-making and elevates the level of patient care. However, a precise definition of conscience in the nursing field is lacking, making it challenging to measure. To address this issue, this study employed the hybrid approach of Schwartz Barcott and Kim to analyze the concept of conscience-based nursing care.

**Methods:**

This approach involves a three-phase process; theoretical, fieldwork, and analytical. A systematic literature review was conducted using electronic databases during the first phase to find relevant papers. The content of 42 articles that met the inclusion criteria was extracted to determine the attributes, antecedents, and consequences of consciousness care using thematic analysis. Based on the working definition as a product of this phase, the plan of doing the fieldwork phase was designed. During this phase, data were collected through interviews with nurses all of whom were responsible for patient care in hospitals. In this phase, 5 participants were chosen for in-depth interviewing by purposeful sampling. Data were analyzed using directed content analysis. The findings of the theoretical and fieldwork phases were integrated and the final definition was derived.

**Results:**

The integration of the theoretical and fieldwork phases resulted in identifying four key characteristics of conscience-based nursing care. Firstly, it involves providing professional care with a conscientious approach. Secondly, ethics is at the core of conscience-based care. Thirdly, external spirituality plays a significant role in shaping one's conscience in this context. Finally, conscience-based nursing care is both endogenous and exogenous, with professional commitment being the central focus of care.

**Conclusion:**

Conscience-based nursing care is an essential component of ethical care, which elevates clinical practice to professional care. It requires the integration of individual and social values, influenced by personal beliefs and cultural backgrounds, and supported by professional competence, resources, and a conducive organizational atmosphere in the healthcare field. This approach leads to the provision of responsive care, moral integrity, and individual excellence, ultimately culminating in the development of professionalism in nursing.

**Supplementary Information:**

The online version contains supplementary material available at 10.1186/s12910-024-01070-8.

## Introduction

Ethics, empathy, and ethical sensitivity are important concepts in nursing that guide the delivery of high-quality care. Ethics refers to the principles and values that govern moral behavior and decision-making [1]. Empathy is the ability to understand and share the feelings of others, while ethical sensitivity is the ability to recognize and respond to ethical issues and conflicts in clinical practice [2]. In the context of nursing, these concepts are essential for delivering conscience-based care, which is care that is guided by the nurse's moral and ethical principles. Conscience-based care is critical in situations where there may be tensions between the nurse's values and the demands of the clinical situation [3].

Nursing revolves around providing care, which has always been integral to nursing definitions [4]. Care in nursing is the goal, mission, essence, moral ideal, and art of nursing [5]. Therefore, care complements the four metaparadigm concepts of nursing and helps determine the nursing discipline. As a moral obligation, it adds a human aspect to the technical duties of nursing [5]. It is important to note that, according to Meleis' definition, the nurse's clinical knowledge and performance alone are insufficient. Clients require a certain level of moral and psychological care for complete recovery [6].

Professional ethics is a fundamental aspect of nursing that encompasses principles, values, and standards [7]. These values are an integral part of care and are reflected in professional ethics codes that serve as guidelines for nurses' care performance. These codes include concepts such as effective communication with colleagues and patients, respecting patients' rights, taking responsibility, upholding dignity, preserving confidentiality, and respecting patients' autonomy in decision-making [8]. In clinical situations, nurses are required to analyze care issues from an ethical perspective and possess the ability to reason and make ethical decisions. They must also have the means of obtaining moral commitment and internalizing moral behavior within themselves. Therefore, it is crucial to understand the strategies for ethical performance and achieving conscience-based satisfaction [9].

Conscience, also known as the inner voice, serves as a moral compass that guides a person's thoughts and actions. It determines what is right and wrong, and can act as a reviewer and judge of past actions, as well as a reference and guide for future actions [10]. The Oxford Dictionary defines conscience as “one’s moral sense of right and wrong, which serves as a guide to one's behavior” [11]. In Islamic texts, various words are used to describe the concept of conscience, including soul and heart, and moral decisions are made with the ultimate goal of achieving God's satisfaction [12].

Conscience holds a significant place in the nursing profession, serving as the foundation and cornerstone of clinical practice. It is defined as the protector of individual integrity, beliefs, and values of the nurse [13]. The nurse's conscience plays a crucial role in guiding professional decision-making and ensuring the provision of high-quality patient care. It has a positive impact on nursing performance and promotes ethical care at the bedside [14]. Conscience also promotes the sense of responsibility among nurses and requires them to utilize their knowledge and skills in providing patient care [15].

In a qualitative study conducted by Jensen et al., nurses described conscientiousness as a motivating factor for taking courageous caregiving actions. When nurses act courageously based on their conscience, they perform their actions in a professional manner, drawing from their experience and knowledge [16]. Providing care can be challenging, and there are situations where nurses must act bravely and follow their inner voice or conscience [17–19]. Such courageous performance based on nurses' conscience can be a strong motivating factor in improving the quality of patient care [19].

In the clinical practice of nursing, conscience can act as a warning system that alerts nurses when their personal and professional values, moral beliefs, and standards are at risk due to problems and challenges they encounter in different situations [20]. When nurses are asked to describe the ethical problems, they face while caring for patients, they often attribute these challenges to their conscience. This inner voice prohibits them from certain actions and commands them to take others [21].

Nurses encounter numerous moral challenges on a daily basis, and they must make moral decisions based on their conscience. Failing to act in accordance with their conscience and violating moral and conscientious values can lead to feelings of guilt, which Glasberg refers to as the conscience problem. These feelings can persist for a long time, even years after the occurrence [22, 23]. When a nurse is repeatedly placed in challenging situations and has a troubled conscience, a phenomenon known as the stress of conscience can arise [24]. Studies have revealed that stress of conscience not only has a negative impact on nurses' professional performance and the quality of patient care, but also affects their personal life, leading to burnout over time. To escape from the stress of conscience, some nurses may even decide to abandon their conscience [25–27].

Research has shown that in caring for patients, especially in special departments and with elderly patients, nurses face obstacles such as lack of time, resources, and organizational barriers that prevent them from providing quality care. This, in turn, creates stress of conscience in nurses, which is strongly and directly related to burnout among healthcare professionals. A study in Iran found that following one's conscience or performing actions based on conscience is the primary source of occupational stress among Iranian nurses in dealing with moral dilemmas and the lack of time and resources during care [28].

In summary, the literature review highlights the importance of conscientious care in nursing, the obstacles to conscientious practice, and the consequences of unconscionable work in the healthcare system. Care based on conscience is viewed as a responsibility-oriented and courage-oriented practice that promotes quality care [16]. When nurses provide care based on their conscience, it is positively perceived, and quality care is delivered [29].

Research shows that the concept of conscience has different conceptualizations in various fields, such as moral philosophy, nursing, and psychology. In the philosophical literature, Heidegger interprets conscience as a silent call to prove human existence, while some arguments suggest that conscience is the voice of God. In psychology, Freud referred to conscience (superego) as a type of integration of parental personality and other skill values, while Frankl believes that there are differences between the superego and the real conscience, and considers conscience to be the core of our existence.

In nursing, Nelms suggests that the call of conscience creates a clear and reliable awareness of patients, their families, and others [30]. According to Dahlqvist et al., conscience in care is described as a driver of personal growth and a protector of personal integrity, which can be used to protect the integrity and dignity of all involved people in care [31]. Furthermore, in studies that have examined difficult ethical situations, care providers have reported that their conscience guides them in performing certain actions, and this guide serves as a motivation to protect patients and their own integrity [26, 32].

The studies and definitions mentioned above have shed light on various aspects of the complex dimensions of the concept of conscience from different perspectives, particularly its theoretical and mental aspects in the context of nursing. However, these definitions do not provide a clear and objective picture of the concept and its dimensions and characteristics. This problem is compounded by the fact that definitions are dependent on the cultural background from which they are derived. This lack of clarity in the definition of the concept can make it challenging to study and measure. Therefore, a detailed examination and analysis of this concept are necessary.

Concept analysis is one method of developing concepts that help to classify and organize nursing experiences, lead to a common interpretation of the concept, prevent personal perceptions and conflicts, and ultimately strengthen the nursing discipline. The hybrid model, proposed by Schwartz-Barcott and Kim (1993), is considered the best method of concept analysis for concepts that are used in a non-transparent manner in the hospital. This model combines both theoretical analysis and experimental observations, making it most applicable to nursing [33].

The hybrid model of Donna Schwartz-Barcott and Hesook Suzie Kim is the concept analysis method used in this study, suitable for situations where the concept being defined is closely connected to practical nursing work. This model has been used as a concept analysis method in the analysis of ethnic concepts in nursing, with various concepts clarified, such as the concept of 'sensitivity' developed and the concept of 'moral courage' analyzed (Schwartz-Barcott & Kim, 1993). In this study, the concept of conscience-based care, along with the determination of its characteristics, antecedents, and consequences, will be explained using the hybrid concept analysis approach. This will lead to a clear definition of the concept and a better understanding of its dimensions and characteristics in the context of nursing [34, 35].

Therefore, in this study, the concept of conscience-based care, along with the determination of its characteristics, antecedents, and consequences, will be explained using the hybrid concept analysis approach. This will lead to a clear definition of the concept and a better understanding of its dimensions and characteristics in the context of nursing.

## Methods

### Study design

The study utilized the hybrid model of concept analysis to examine the characteristics of Conscience-based Nursing Care. This approach, introduced by Schwartz-Barcott and Kim in 1986, is a method of conceptualization and theoretical development [36, 37]. The hybrid model consists of three phases: theoretical, fieldwork, and analytical [35]. In the theoretical phase, the literature was systematically reviewed to establish the essence of the concept, including its definition and measurement. The fieldwork phase involved collecting qualitative data in a real setting to verify and improve the concept's attributes. In the analytical phase, the data from the previous phases were analyzed and compared to further refine the concept's definition [35]. The stages of the hybrid model—theoretical, empirical, and analytical—while occurring in a sequential manner, are not disconnected. Instead, there are instances of overlap where learnings from one stage feed into the other. They build on each other, creating an iterative cyclical paradigm. Our aim was to define the Conscience-based Nursing Care based on a fusion of a broad literature review and the integration of empirical data collected directly from the field.

#### Theoretical phase

The initial phase of the study, which constitutes a systematic literature review, encompasses a series of methodologically rigorous steps. These include selecting the concept, conducting a comprehensive literature review, and elaborating and refining the concept’s definition. The purpose of this phase was to deepen the understanding and elaborate the definition of Conscience-based Nursing Care by systematically examining existing scholarly work. A diverse set of international databases were searched including PubMed, Web of Science, Scopus, and Google Scholar, alongside national databases such as SID, MAGIRAN, IRANDOC, and Iranian Database of Medical Literature (IDML). This search spanned literature from the concept’s inception in 1978 through to 2020. The search parameters were limited to articles published in English. Keywords and their synonyms, such as “Conscience”, “Conscience in nursing”, “Conscience in Nursing Practice”, “Perceptions of conscience”, “Stress of Conscience”, and “Conscientious objection”, were methodically applied. The central questions guiding the search were: “How Conscience-based Nursing Care is described, defined, and operationalized?” and “What constitutes the attributes of Conscience-based Nursing Care?” (Search Stratgy Supplement 1).

We included original and review articles that addressed at least one aspect of the search queries—whether descriptive, definitional, or attributive to Conscience-based Nursing Care. Concurrently, we excluded not only letters to the editor and commentaries but also any publications that were not peer-reviewed, abstracts from conferences, case reports, and articles that did not focus explicitly on the topic of Conscience-based Nursing Care.

### Study selection process

**Fig. 1:**
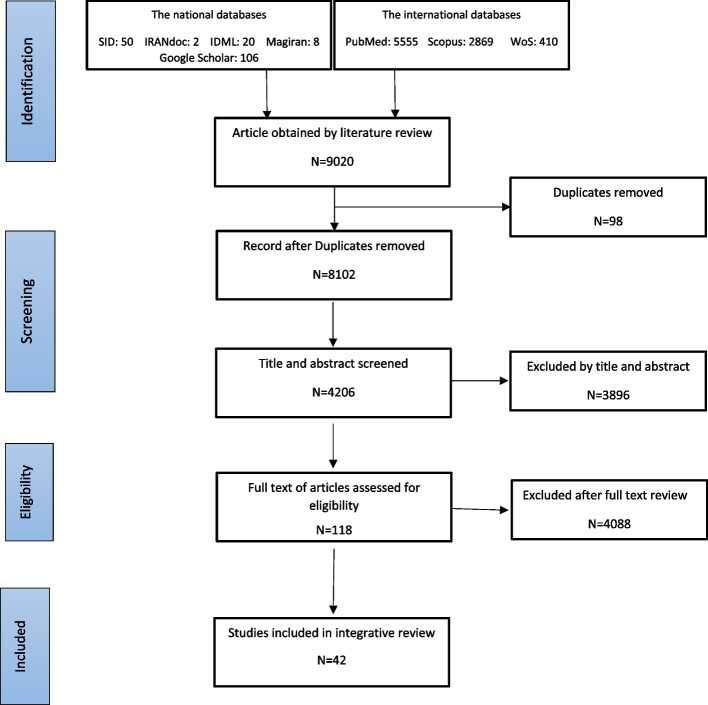
Literature selection process

Articles were initially retrieved and imported into EndNote software for the elimination of duplicates during the designated data collection period, which spans from January 1978 to December 2020. The full texts of articles that met the inclusion criteria were then reviewed. Data selection and extraction were performed independently by two researchers to uphold methodological integrity. Discrepancies encountered during this process were resolved through discussion or, if necessary, third-party adjudication. Figure 1 illustrates the study selection process: in the theoretical phase, 9020 abstracts were screened over the data collection period and were eventually narrowed down to 42 articles for in-depth analysis, as detailed in Table 1.

**Table 1:** Selected articles for a comprehensive literature review

	**Title**	**Authors**	**year**	**Methodology**	**Country**
1	Assessments of stress consciousness, perceptions of consciousness, burnout, and social support before and after implementation of a participatory action-research-based intervention	Ericson-Lidman, Ahlin [38]	2017	Participatory Action Research	Sweden
2	Care providers learning to deal with troubled conscience through participatory action research	Ericson-Lidman, Strandberg [39]	2013	Participatory Action Research	Sweden
3	The intuitive nurse in critical care practice? A phenomenological study	Hassani et al. [40]	2016	Phenomenology	Iran
4	Conflicts of conscience in the neonatal intensive care unit: perspectives of alberta	Ford, Austin [41]	2017	Interpretive Description	Canada
5	Levels of consciousness and related factors among iranians Oncology nurses	Gorbanzadeh et al. [42]	2015	Descriptive Correlational	Iran
6	Compassion and responsibility in surgical care	Torjuul et al. [43]	2007	Phenomenology	Norway
7	Personal conscience and the problem of moral certainty	Vaiani [44]	2009	Review	USA
8	The influence of conscience in nursing	Jensen et al. [16]	2009	Phenomenographic	Sweden
9	Factors affecting conscience-based nursing practices: a qualitative study	Jasemi et al. [3]	2019	Qualitative Content Analysis	Iran
10	Nurses' strategies for consciousness-based care delivery: a qualitative study	Jasemi et al. [15]	2019	Qualitative Content Analysis	Iran
11	Perceptions of conscience in relation to stress of conscience	Juthberg et al. [45]	2007	Descriptive Correlational	Sweden
12	Burnout and perceptions of conscience among health care personnel: a pilot study	Gustafsson et al. [26]	2010	Descriptive Correlational	Sweden
13	Conscientious objection in nursing: definition and criteria for acceptance	Lachman [46]	2014	Review	USA
14	Healthcare practitioners' experiences of postoperative pain management in lumbar spine surgery care—a qualitative study	Angelini et al. [47]	2020	Descriptive Qualitative Study	Sweden
15	Ethically difficult situations in hemodialysis care – nurses' narratives	Fischer Grönlund et al. [25]	2015	Phenomenological Hermeneutic Approach	Sweden
16	Nurses' ethical reflections on caring for people with malodorous exuding ulcers	Lindahl et al. [48]	2010	Qualitative Content Analysis	Sweden
17	Yes we can! Successful examples of disallowing 'conscientious objection' in reproductive health care	Fiala et al. [49]	2016	Investigating The Laws And Experiences	Austria, Sweden, Finland
18	Care providers' experiences of guidelines in daily work at a municipal residential care facility for older people	Åhlin et al. [50]	2014	Qualitative Descriptive	Sweden
19	Nurses' use of conscientious objection and the implications for conscience	Lamb et al. [51]	2018	Interpretive Phenomenology	Canada
20	Conscience, conscientious objection, and nursing: a concept analysis	Lamb et al. [10]	2019	Concept Analysis	Canada
21	Meanings of troubled conscience and how to deal with it: expressions of persian-speaking enrolled nurses in sweden	Mazaheri et al. [52]	2018	Phenomenological Hermeneutic	Sweden
22	A threat to our integrity – meanings of providing nursing care for older patients with cognitive impairment in acute care settings	Nilsson et al. [53]	2016	Phenomenological Hermeneutic	Sweden
23	Moral courage in nursing: a concept analysis	Numminen et al. [19]	2017	Concept Analysis	Finland
24	Questions of conscience	Salladay [54]	2017	Review	USA
25	Duty and dilemma: perioperative nurses hiding an objection to participate in organ procurement surgery	Smith [55]	2017	Qualitative Grounded Theory	Australia
26	“Conscience” clauses the rights and responsibilities of a nurse	Tillett [56]	2018	Review	USA
27	Opinions of nurses regarding conscientious objection	Toro-Flores et al. [57]	2017	Cross-Cutting Descriptive Study	Spain
28	Nurses' experiences of busyness in their daily work	Govasli, Solvoll [58]	2020	Phenomenological Hermeneutical	Norway
29	Clear conscience grounded in relations: expressions of persian-speaking nurses in sweden	Mazaheri et al. [59]	2016	Phenomenological Hermeneutical	Sweden
30	Meeting ethical challenges in acute care work as narrated by enrolled nurses	Sørlie et al. [60]	2005	Phenomenological Hermeneutical	Sweden
31	The meaning of being in ethically difficult care situations in pediatric care as narrated by female registered nurses	Sørlie et al. [61]	2003	Phenomenological Hermeneutical	Sweden
32	Slovak healthcare workers' lived experience of conscience	Blaho [62]	2016	Phenomenological Hermeneutical	Slovakia
33	The role of consciousness in nursing practice	Cleary, Lees [20]	2019	Review	Australia
34	A comparison of assessments and relationships of stress conscience, perceptions of conscience, burnout and social support between healthcare personnel working at two different organizations for care of older people	Ahlin et al. [63]	2015	Cross-Sectional, Descriptive Comparative	Sweden
35	Nurses lived experiences of conscience reaction: a qualitative phenomenological study	Hasani et al. [64]	2011	Phenomenological Hermeneutic	Iran
36	Experiences of gynecologic oncology nurses regarding caring behaviors: a hermeneutic phenomenological study	Boz, Teskereci [65]	2020	Phenomenological Hermeneutic	Turkey
37	Ethical diversity and the role of conscience in clinical medicine	Genuis, Lipp [66]	2013	Review	Canada
38	Nurses' experience of the perception of nursing consciousness: a phenomenological study	Hasani et al. [67]	2013	Phenomenology	Iran
39	Development of the perceptions of conscience Questionnaire	Dahlqvist et al. [68]	2007	Psychometric	Sweden
40	Psychometric properties concerning four instruments measuring job satisfaction, strain, and stress of conscience in a residential care context	Orrung Wallin et al. [69]	2013	Psychometric	Sweden
41	Stress of conscience questionnaire (scq): exploring dimensionality and psychometric properties at a tertiary hospital in australia	Jokwiro et al. [70]	2020	Cross-Sectional Study	Australia
42	Development and initial validation of the stress of conscience questionnaire	Glasberg et al. [71]	2006	Psychometric	Sweden

A directed content analysis approach was employed for data analysis. Multiple readings of the articles aided in distilling relevant codes, which were then synthesized, integrated, and categorized into antecedents, attributes, and consequences. The research team diligently searched for terms associated with Conscience-based Nursing Care, grouping resultant codes into coherent categories.

Following a critical comparison of existing definitions in the literature with the initial definition, a refined definition was developed. This refined definition took into account the multidimensional characteristics of the concept and was couched in nursing-specific terms. It was this definition that provided a foundation for the subsequent fieldwork phase.

Additional exclusion criteria, beyond the non-inclusion of commentaries and editor’s letters, included the omission of non-peer-reviewed articles, unpublished theses or dissertations, and conference abstracts. Such criteria ensured the selection of robust, peer-reviewed empirical and theoretical works that meaningfully contributed to the study’s overarching research objectives.

#### The fieldwork phase

During the fieldwork phase of our study employing a hybrid model, qualitative data were gathered through one-on-one interviews, with a focus on in-depth concept analysis. The literature continued to be reviewed throughout to align the freshly collected empirical data with existing knowledge. The in-depth interviews were specifically designed to integrate the concept analysis from the theoretical phase with real-world empirical data, enhancing our comprehension of the nursing care concept by conscience [36, 72].

In accordance with Schwartz-Barcott & Kim’s original hybrid model from 1993, the empirical phase primarily consisted of observational data [34, 35]. However, in keeping with more recent methodologies, we expanded upon this to encompass additional qualitative data collection techniques, such as one-on-one interviews. This diversification of empirical sources was valuable to add breadth and depth to our research. The qualitative, empirical data generated through these techniques served as a crucial reference point, enriching and refining the theoretical definition derived in our theoretical phase.

These qualitative interviews allowed us to holistically understand the phenomenon under study by providing real-life contexts and insights. Consequently, it lent substantive strength to our theoretical definitions and proposed a new perspective, especially concerning the nuanced meaning of the studied concept.

The interviews were conducted by two independent researchers whose familiarity with the field of conscience-based nursing care was established through rigorous training, and their integration into the field was facilitated through preparatory visits to participating clinical departments. This allowed for establishing rapport without prior relationships influencing the responses. The researchers had no previous direct contact with the participants before the study, eliminating bias from pre-existing opinions or experiences.

These interviews were conducted by two researchers with backgrounds in nursing research; neither had any prior contact with the participants nor were they part of the participants’ hospital teams. This assured that the data collected were uninfluenced by pre-established relationships. The researchers were incorporated into the clinical setting as observers prior to data collection to familiarize themselves with the environment, yet maintained a professional distance to minimize observer bias.

The concept of nursing care guided by conscience was explored with participants hailing from various departments within the medical education centers affiliated with Golestan University of Medical Sciences. Participants represented diverse demographics and were chosen based on characteristics identified in the literature review to ensure comprehensive representation. Each participant provided informed consent, affirming their willingness and ability to share detailed experiential data.

Interviews took place in the clinical units of the participants, with durations respecting the participants’ willingness, ranging from 30 to 45 min. The saturation point guided the extent of data collection, which was deemed as reached when additional interviews no longer yielded novel insights. Our semi-structured interview approach utilized an interview guide with open-ended questions derived from theoretical constructs identified earlier. The guide, enclosed as a supplementary file, was assessed through a pilot study involving five nurses not involved in the main study. Minor adjustments followed this pilot to refine the interview questions. Examples of questions include descriptions of workdays, decision-making in difficult situations, and instances where care aligns with personal conscience.

Clarifying and exploratory queries, such as “Could you elaborate more on that?”, supplemented the interviews to ensure comprehensive understanding and to delve deeper into the phenomena. We ensured to offer participants the opportunity to share any additional thoughts at the interview’s conclusion. The data were subjected to a rigorous analysis facilitated by a team of three coders using a five-step qualitative content analysis method with intersubjective verification of the coding process.

The collected data were analyzed using a five-step qualitative content analysis method [73], which involved: (I) Transcribing the recorded interviews, (II) Reviewing the transcripts to gain a general understanding of the content, (III) Identifying meaningful units and primary codes, (IV) Classifying the primary codes into broader categories, and (V) Specifying latent themes. In this study, the recorded interviews were transcribed verbatim immediately after each interview. In the second step, the transcripts were carefully reviewed, and the text was divided into meaningful units. In the third step, meaningful units were abstracted and coded using MAXQDA software (version 2010) to identify explicit and implicit concepts from the participants' experiences and statements. The codes were then summarized and classified into categories based on similarities, differences, and relevance. In the final step, themes were formulated to express the latent content of the text.

### Analytic phase

In the analytic phase, the findings from the theoretical analysis and fieldwork phase were combined and analyzed together to arrive at a comprehensive and clear definition of the concept [35]. This involved integrating the theoretical and fieldwork findings to determine the antecedents, attributes, and consequences of the concept, and to provide a new definition. During the final analytical phase, the attributes, antecedents, and consequences identified in both the theoretical and fieldwork phase were integrated to create a new definition for the concept.

### Rigor

To ensure the rigor and trustworthiness of the data, four criteria proposed by Guba and Lincoln were used, which included credibility, dependability, confirmability, and transferability [74]. Before starting the study, a few pilot interviews were conducted to gain an initial understanding of the subject matter. To enhance the dependability of the extracted codes, they were reviewed by some participants and modified according to their feedback. To check the confirmability of the findings, they were peer-checked by experts, and the findings were revised according to their opinions. To enhance the transferability of the data, participants were selected with maximum variation, and the research procedure and findings were reported in detail to allow for replication and comparison. Overall, these measures were taken to ensure the rigor and trustworthiness of the data.

## Results

### Theoretical phase

#### Definition of conscience in other academic disciplines and religions

The retrieved articles were analyzed using content analysis, which involved forming categories based on the general characteristics of the articles. A table was created to summarize the findings of the analysis. The results of the analysis provided a detailed understanding of the content of the articles. The term “conscience” has its roots in the Latin word “conscientia” which refers to sharing knowledge, awareness, and understanding. In English, “conscience” is defined as one's moral sense of right and wrong, serving as a guide to one's behavior. It can also refer to one's inner self, intelligence, and the nature of existence. In Persian, “conscience” is defined as the soul and inner powers that enable a person to discern good from bad actions.

The concept of conscience has been interpreted differently across various schools of thought and religions. A review of literature has uncovered conflicting views on conscience in moral philosophy, theology, psychology, and nursing. The origins of conscience have been attributed to different sources such as God, society, and the individual self. Moreover, the nature of conscience has been the subject of debate, with some emphasizing reason, others emotion, and still others personal integrity.

To illustrate, Heidegger views conscience as the call of Dasein in philosophical and theological literature. In natural law, conscience is believed to be the voice of God in human existence. Theological literature also associates conscience with the voice of God [75]. Frankel regards conscience as the essence of our existence [76]. In Abrahamic religions, conscience is derived from ethical principles prescribed and supported by God and passed down through tradition. In Western Christian traditions, conscience is rooted in the Greek word “syndesis” and relates to civil responsibility, common sense, and special action. In Islamic texts, various parallel words indicate the concept of conscience, such as soul and heart, and moral decisions are guided by the ultimate goal of pleasing God. The Quran interprets the concept of conscience subtly, depicting man as having natural and conscientious inspirations and perceptions that are not acquired from any external source. This concept is reflected in verses related to “soul”, “heart”, “conscience”, and “nature” as well as traditions with similar themes. The Quran also references the “awakened soul and awakened conscience” and highlights the authority of the soul in various verses. In instances where the soul is not subject to reason, it is referred to as the “imperious soul”.

#### Definition of conscience in nursing

Conscience has been a central focus in the nursing profession since Florence Nightingale, and it is considered a key personality trait related to professional competence. It promotes nurses' sense of responsibility and requires them to use their knowledge and skills in providing patient care. Conscience guides nurses in determining what is right in their clinical practice and ultimately influences their decision-making. It plays a critical role in providing accurate and reliable nursing care.

Conscientiousness is a valuable asset that guides nurses in their efforts to provide high-quality care. Additionally, conscience is a fundamental ethical concept in nursing and is considered a cornerstone of ethical care, having a positive impact on nurses' ethical practice. Cleary emphasizes the importance of conscience in a person's moral integrity and defines it in the nursing profession as a mental process that strives for the originality and integrity of the individual based on their best ability to make moral choices [20]. Therefore, conscience can be seen as a warning system that alerts us to potential threats to our personal and professional values, beliefs, morals, or standards when faced with challenging issues or circumstances.

#### Features of conscience-based nursing care

Through literature review and content analysis, two categories of attributes of conscience-based nursing care were identified: (1) Ethics in Care and (2) Individual and Social Beliefs and Values. These categories and their corresponding subcategories are presented in Table 2, along with some concepts from the studies that were reviewed.

**Table 2:** Attributes related to the concept of conscience-based nursing care

Categories of attributes of the concept	Subcategories of attributes of the concept	Some concepts obtained from studies
Ethics in care	- Manifestaion of professional ethics - Direction care	- Conscience is a concept in the field of medical ethics [71] - A good conscience is associated with feelings of integrity and ethical responsibility [31] - The conscience is an inner guide grounded in feelings [52] - Conscience is an asset that guides nurses in their efforts to provide high quality care [16]
Individual and social beliefs and values	- Arising from a religious and cultural background - Reflecting the integrity of individual values - inner voice	- Affected by culture, conscience is a valuable component of nursing practice which demands sensitivity [68]. - Conscience is formed and affected by sociocultural and religious beliefs [4, 5] - Conscience is the mental process that strives to maintain an individual's authenticity and integrity by alerting the individual to potential violations of his or her values… [77] - By the inner calling, we mean the voice of conscience which draws nurses' attention to all patients' needs and directs them towards quality conscience—based care delivery [3]. - Conscience is described as an inner voice from God [75]

#### Antecedents of conscience-based nursing care

The antecedents or factors that precede the concept of conscience-based nursing care include (1) Organization, with two subcategories: sources and supportive atmosphere; (2) Professional Competence, with four subcategories: attitude, knowledge and professional skills, communication with patients and companions, teamwork, and ethical commitment; and (3) Personality Traits, with two subcategories: individual abilities and individual values and beliefs. These antecedents and their corresponding subcategories are listed in Table 3.

**Table 3:** Antecedents related to the concept of conscience-based nursing care

Categories of antecedents of the concept	Subcategories of antecedents of the concept	Some concepts obtained from studies
-Organization	- References - Supportive atmosphere	- One of the factors that nurses frequently express as the leading cause of refusing conscience-based nursing practices is the high workload [3]. - Experience and time acted as a source of guidance and helped the nurses gain confidence in their nursing actions at times of a conflict of conscience [41] - The novice nurses need a massive support from colleagues and managers. Providing adequate support for novice nurses reduces stress and supplies a platform to deliver conscientious nursing care [3]. - A person's identity is dependent on another person in a good or bad relationship, as well as on a voice from within the person, her or his conscience. Confirmation is about both social confirmation from another person and self-confirmation from one's own conscience [61] - In supportive environments where the nurse feels well supported to voice their concerns and feelings they are often more likely to lower or manage their stress levels associated with their conscience [26]
Professional competence	- Attitude, knowledge and professional skills - Communication with the patient and team work companions - Ethical commitment	- Endeavoring to do the daily nursing tasks in the proper way contributes to keeping the conscience clear [52] - In nursing profession, conscience is considered as a personality—related component of professional competence which promotes nurses' sense of responsibility and requires them to use knowledge and skills in patient care delivery [68] - Nurses identified professional commitment and responsibility as the key element in conscience-based nursing practices [3]. - The ethical and professional principles of care play an important role in enhancing professional conscience [3]. - Nurses' effective communication with patients and colleagues has key roles in the identification of patients' needs and the delivery of conscience—based care [15] - Achieving integrity of conscience is also linked to a professional and humanistic approach to the patient [62].
Personality characteristics	- _ _ _ _Individual abilities - _ _ _Individual values - _Individual beliefs	- Practical and theoretical learning was strategy for professional conscience—based care delivery [15] - Nurses declared that religious beliefs may direct the conscience-based practices in performing optimal care. Planning and implementing a comprehensive care requires recognition of patients' needs which might be influenced by religious beliefs [3] - The conscience develops in childhood and is subject to growth during life. It is learned mainly from parents who also learned from previous generations [59]

#### Consequences of conscience-based nursing care

Through text analysis in the theoretical phase, it was determined that the consequences of conscience-based nursing care include responsive care, promotion of professional ethics, and emotional well-being. Table 4 provides further details on each of these consequences.

**Table 4:** Consequences related to the concept of conscience-based nursing care

Categories of consequences of the concept	Subcategories of consequences of the concept	Some concepts obtained from studies
Responsive care	- Responsible care - Understanding patient needs	- Individual conscience acts as a promoting factor to perform appropriate nursing practices while prohibiting wrong practices [5–8]. - Conscience promotes nurses' responsibility and accountability towards medical errors. In addition, it helps nurses to identify client's demand and enable them to develop care plan [9].
Promotion of professional ethics	- Moral integrity - Moral courage - Patient rights	- Conscience was the driving force behind courageous acts, giving courage to discuss difficult subjects. Conscience strengthened nurses' ability to stick to their values and set boundaries to their actions. It helps to question prevailing practices and opinions [19]. - Conscience is a prerequisite to ethical behavior and has an important role in providing accurate and secure nursing care [68] - Conscience as an “act of high quality care” nurses promote patients' rights to receive quality care believing that nurses are responsible for ensuring this [16]
emotional consequences	- Positive emotions - Negative emotions	- Promotion of conscience—based practice in nursing helps nurses closely adhere to ethical standards, develops their professional roles and justice seeking, and gives them feelings of calmness, happiness, satisfaction and inner peace [1, 7]. - Nurses' experiences of powerlessness also meant that they had to deaden their conscience [48]. - If we do not follow conscience, blaming and expostulation phenomenon seen clearly. Clearance and consistency of this phenomenon is more than other consciousness phenomena. Absolutely blaming is the aspect of conscience that protects our ideal personality, and promotes it more than ever is an antecedent for psychological development. This phenomenon is the greatest humanistic condition that human being has a clear conscience. These people benefit from all conscience activities [64]

#### The working definition of conscience-based nursing care

The operational definition of conscience-based nursing care in this study is as follows: “Conscience-based nursing care is the provision of care based on responsibility, clinical competence, and moral integrity in the healthcare field, which leads to improved quality of care, patient satisfaction, and personal and professional excellence”.

Here are two examples that illustrate the model as identified through the literature review:

##### Model case

Mrs. S.P. is an emergency department nurse at a trauma center. She arrived on time for her shift with a tidy and professional appearance and greeted her colleagues before actively participating in the shift handover. Mrs. S.P. took responsibility for the four patients in the emergency trauma department and introduced herself to each patient, encouraging them to let her know if they needed anything. She thoroughly examined each patient, identified their needs, and provided comprehensive care. Despite being informed by the previous nurse that all patients were stable, Mrs. S.P. re-checked each patient's vital signs using the Glasgow Coma Scale and the Revised Trauma Score. During blood circulation monitoring of a patient with a closed ankle fracture, she noticed the absence of a pulse and cool skin temperature and immediately informed the emergency medicine doctor. After coordinating with the doctor and supervisor, the patient was transferred to the operating room for further treatment. Mrs. S.P. maintained the patient's privacy and provided realistic hope based on medical history. She also noticed another patient with head injuries and promptly informed her colleagues and supervisor, receiving quick assistance. Mrs. S.P. continued to provide care for her remaining patients, documenting her care in a legible and honest manner. At the end of the shift, she double-checked her duties and informed her patients of her departure, leaving a positive impression on them. The superintendent commended Mrs. S.P. for her thoroughness, and her patients were grateful for her care and kindness.

##### Opposite case

Mr. S.R. works as a nurse in the surgical department of a medical training center. He arrives for his morning shift a little late and appears confused. When asked about the delay, he blames traffic. After receiving a handover from his colleagues, he is assigned to care for five post-surgery patients by the supervisor. At the beginning of his shift, Mr. S.R. goes to the nursing station with the patients' files and begins writing his nursing reports. While checking the files and writing reports, he instructs a field student to perform tasks for the patients without considering the student's knowledge and skills. These procedures include changing dressings, checking drains, and fixing Foley catheters. Mr. S.R. calmly checks his cell phone messages and registers the patients' medication. When the student asks to accompany him, Mr. S.R. responds unenthusiastically and proceeds to the patient's bedside. He does not greet or explain anything to the patient before lifting her clothes to change the dressing, disregarding her privacy. The patient is distressed but does not say anything. Mr. S.R. declines to wear gloves, stating that he is sterilized, but the patient questions this practice. Despite her pain and redness and discharge at the surgical site, he insists on changing the dressing and threatens not to do so again if she resists. After completing the task, he leaves the equipment for the student to collect. He performs catheterization for another patient without following standard procedures, such as wearing sterile gloves and lubricant gel. He also fails to take the patients' vital signs accurately. At the end of the shift, he leaves the ward without sufficient explanation. None of his colleagues or patients are satisfied with his behavior.

### Field phase

Table 5 provides the demographic characteristics of the 5 participants (3 women and 2 men) who were included in the present study and were at the working stage in their respective fields.

**Table 5:** Characteristics of the participants

Participant	Age	gender	Marital statue	work experience	The current clinical department	Education level	position
1	36	Female	married	13	emergency	Masters	In charge of the rotating shift
2	51	Female	married	27	Burn emergency	Masters	Superintendent
3	28	Man	Single	5	ICU	Masters	Night shift manager
4	33	Female	married	11	Pediatric surgery	Masters	Nurse
5	40	Man	Single	13	Orthopedic surgery	Masters	Nurse

The extracted features of the concept of conscience-based care in the stage of work in the field. From the participants' interviews, four main themes were identified as the characteristics of conscience-based care during the working stage in the field. These themes are: “ethicalism as the foundation of conscience-based care,” “professional care as a manifestation of conscientiousness”, “spiritual and educational beliefs”, and “care centered on professional commitment”. The theme of ethicalism in conscience-based care has two sub-themes: “stimulating moral sensitivity” and “encouraging ethical courage”. The theme of professional care as conscientious care has three sub-themes: “scholarly care”, “humanistic care”, and “client-centered care”. The main theme of educational and spiritual beliefs has three sub-themes: “family education”, “spiritual beliefs”  and “religious beliefs” Finally, the theme of care centered on professional commitment has a subset of “professional identity” and “service motivation” as shown in Table 6.

**Table 6:** Features of the concept of Conscience-based Nursing Care based on fieldwork

Main t theme	Sub-themes
Moralism at the heart of conscientious care	Arousing moral sensitivity
	Moral courage
Professional care as conscientious care	Scientific care
	Customer-centric
	Humanistic care
Spiritual and educational beliefs	Family education
	Spiritual beliefs
	Spiritual beliefs
The nature of the profession	Knowledge of the profession
	Motivation to serve

Ethicalism is a fundamental aspect of conscience-based nursing care, as it is one of the main themes that emerged from nurses' experiences. Moral courage is considered a defining characteristic of conscience-based care. Nurse number 5, for example, stated that her conscience has often given her the strength to advocate for patients when faced with incorrect opinions from doctors, colleagues, or supervisors. In her view, it is unethical to remain silent when something is amiss in patient care, as doing so would lead to feelings of guilt.

Based on the participants' experiences, it appears that professional care is a key characteristic of conscience-based care. Nurses who practice conscience-based care strive to integrate scientific, humanistic, and client-centered care approaches in their clinical practice. This combination of care approaches allows nurses to provide a high level of care that is both evidence-based and compassionate, while also focusing on the unique needs and preferences of each patient.

Participant 2 also highlighted how providing correct care ensures a clear conscience. He stated: “It all comes down to helping the sick. Look at a patient who is completely unstable with widespread electrolyte disorders, and then they become so stable. It means I did my job correctly, step by step, and before that my conscience is clear and of course, I am very satisfied with myself.”

He further emphasized that caring for clients regardless of circumstances is part of conscience-based care. He remarked: “It does not matter to me, for example, this patient was complaining so much, again I take care of him, but fatigue leaves one's body, they don't know how to thank me (laughing), my conscience does not allow me to differentiate between patients.

“Participant 5 emphasized the importance of providing accurate and sufficient information to clients. He mentioned a situation involving a child with urological issues. He recounted: “A few years ago, a child had a problem with urology. We talked to a specialist at our facility. At first, the specialist said he could perform the procedure, then later said he could not. A colleague's father was on a flight. The patient arrived at 2:30 in the afternoon. We quickly approved it and he went to Tehran. He went to Hazrat Ali Asghar Hospital. Around 5–6 in the afternoon, the procedure was done and the patient improved. Later, his father came back and thanked us. My conscience was clear that I had guided him correctly”. Participant 5 highlights how ensuring clients receive proper guidance brings a sense of fulfillment.

Participant 4, a nurse with experience in pediatrics and neonatology, highlighted how empathy and putting oneself in patients' situations shapes her conscience-guided care. She remarked: “I have repeatedly mentioned my conflict of conscience in performing the care resulting from twinning with the mothers of the children under my care. I had given birth and I had the same situation, which worries me more. I think of it as your own child. If it was your own child, what would you do for him? Imagine that you have a baby yourself. She is a mother. What should you do for him now in that situation? That's when your conscience gets involved”.

One of the main themes that emerged from the participants' perceptions is care centered on professional commitment, which is considered a defining characteristic of conscience-based care. Having a strong professional identity and motivation to serve is crucial for nurses in providing conscientious care in clinical practice. This helps to ensure that they are able to deliver high-quality care that is grounded in a deep commitment to their profession and their patients.

Participant 2 remarked: “The new nurses have no conscience or morals at all, and another thing is that everyone thinks that they don't like coming to this field, just because they have money. take it. In general, this model of working with new nurses is more common in the new generation”.

Participant 1 shared: “My father told me: 'Son, you are going to study in this field, it is a difficult field, but you are dealing with a bunch of painful people, so concentrate, do your work properly, never lose heart,' that's what my father said when I was going to university. He told me, don't lose your mind, and always keep your conscience awake”.

#### Introduction to the concept of conscience-based care

The study's interviews with participants revealed three main themes that influence conscience-based care: the work environment, professional competence, and institutionalization of values and beliefs. Table 7 summarizes these antecedents.

**Table 7:** Antecedents of the concept of conscience-based nursing care based on conscience in the fieldwork

Main t theme	Sub-themes
Conscientious care affected by work atmosphere	Providing resources
	Supportive interactive atmosphere
Professional competence	Knowledge-oriented and professional skills training
	Ethical commitment
	Development of individual skills
	Professional experience
	Paying attention to academic training
Institutionalization of values and beliefs	Value-oriented effectiveness

One of the important requirements for nurses to provide conscientious care is a supportive work atmosphere, which has two sub-topics: providing resources and fostering a supportive interactive atmosphere. Participants' experiences indicate that having sufficient and skilled personnel, access to necessary medications, adequate time to provide care, and well-planned and organized shifts are necessary prerequisites for providing care based on conscience.

Nurse participant #1 describes an experience of improperly administering intravenous medication to outpatients in a rushed and haphazard manner due to being overwhelmed by a high workload during the COVID-19 pandemic. Specifically, the nurse says:

“For outpatients, I hastily administered various intravenous medications by injecting them directly into the IV line. Of course, the situation was even more dire during the COVID-19 crisis. The doctor ordered me to administer a number of medications intravenously. After giving the patient pentazole intravenously, I proceeded to inject all the other medications directly into the IV line. My colleague told me, “Slow down, you have to administer these separately. Come on, inject them all into my IV!” So I injected everything into the IV line. You won't believe that afterwards, I asked my colleague why we did such a thing. I felt very remorseful, perhaps because with so little time and so much work, I didn't know what decision to make at that moment. Imagine having 100 patients, all needing various medications (serum, Apotel, vitamin C, Ketorolac, B complex, Pantoprazole, and sometimes hyoscine). I knew Hyosin should not be given intravenously because it can cause severe side effects, including extreme dry mouth. However, because I had to empty the beds, the lack of resources and space led me to override my better judgment. In short, the lack of time, staff, and space caused me to violate my conscience. Do you understand what I'm saying?”

One of the main antecedents of conscience-based care is paying attention to equal educational opportunities. Nurse number 3 shares a memory related to this, saying, “I remember during my internship, a suicidal patient was brought to the emergency ward. The ward was very busy, and finding an empty bed was challenging. The patient had taken rice pills, and I had no prior knowledge of such patients. I planned to tend to the patient after completing other tasks, but I couldn't make it on time. I don't even remember why my colleagues didn't attend to him on time; perhaps he was sleeping far from the station, and I don't know. Anyway, the patient died, and I still wonder if I was responsible for his death. (Silence) The reason was the lack of knowledge and education. Shame on these schools that do not provide us with proper training. All these deaths are on the necks of these uneducated professors”.

Another antecedent that contributes to conscience-based care in the workplace is the institutionalization of values and beliefs, which are essential for the formation of such care. Nurse number 4 emphasizes the role of family upbringing and the environment in shaping the function of the conscience. They state, “I also believe that our conscience is influenced by the family environment and atmosphere in which we grew up. In my family, we were always taught to prioritize the well-being of others and to be helpful. These values have had a significant impact on me and my work”

Another nurse shares their perspective on the importance of family upbringing in shaping their approach to care. They state, “I learned about kindness from my father. He taught me that everyone deserves respect, regardless of their background or ethnicity. Even if someone disrespects me, I have no expectations, and I will still treat them with respect. It all depends on how we were raised. It's like planting a sapling, if you plant it crookedly, the tree will grow crooked, and the rain will hit the ground unevenly. But if you plant it properly and water it, it will grow straight. The same principle applies to human beings” (P. 5).

#### Consequences of the concept of conscience-based care

Consequences of the concept of conscience-based care is presented in Table 8.

- Nurse 1: I always fulfill my job responsibilities regardless of the situation. Even if it goes against my conscience, I make sure the system functions properly.
- Nurse 2: I do not differentiate between patients based on who they are. I treat all patients as human beings and carry out my job responsibilities dutifully guided by my conscience.
- Nurse 3: Dealing with an agitated patient who continues to demand more despite receiving the best care can be frustrating. Although it pains my conscience, I end up distancing myself and just doing the minimum required to fulfill my duty as their constant demands become unbearable.
- Nurse 4: As a student nurse, I refused to administer suppositories to a feverish patient with low consciousness because the patient was dirty. I had never disclosed this incident to anyone before.
- Nurse 5: We had a heart patient whose ECG was deemed useless by the general practitioner. I noticed the patient had an arrhythmia. I urgently attended to the patient, started the Acute Coronary Syndrome protocol, cannulated a vein, and provided oxygen therapy and cardiac monitoring. The specialist confirmed it was an arrhythmia. I was satisfied with my decision and my conscience was clear that I had done the right thing.

**Table 8:** Consequences of the concept of conscience-based nursing care based on conscience in the fieldwork

Main theme	Sub-themes
Responsive care	Committed professional care
	Equality of clients
	Empathic communication
	Ignoring the creditor actions of the patient and the patient's companion
Promotion of professional ethics	Ethics in care
Satisfaction of patients and companions	Peace of mind
	Reduce worry and anxiety
emotional consequences	Positive emotional consequences
	Negative emotional consequences

### Analytic phase

In this phase, we undertook a thorough synthesis of findings gathered from the comprehensive literature review and empirical fieldwork data. The objective was to distil these diverse sources of knowledge into a cohesive and concise definition of ‘conscience-based nursing care’. The empirical data highlighted several key attributes of nursing care that were accentuated by nurses’ individual experiences. These encompassed prioritizing professional commitment, leveraging personal professional experience, focusing on robust academic training and operations, and striving for optimal patient satisfaction.

**Fig. 2:**
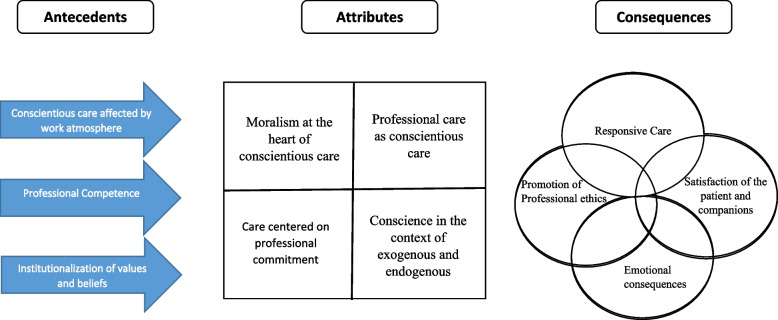
Attributes, antecedents, and consequences of Conscience-based Nursing Care in the final analysis phase

When aligned with the theoretical elements previously identified, including the integral role of conscience in professional care, the underlying moralism of a conscience-centric approach, endogenous and exogenous spirituality in nursing, and an unwavering commitment to care quality, a more refined view emerges (as depicted in Fig. 2).

The concept of encapsulates a multifaceted perspective on the compassionate professionalism within care contexts, grounded in a rich tapestry of ethics and emotional intelligence. Core Professional Ethics and Values are central, emerging from both literature and qualitative research as a combination of ethical practice, personal values, and emotional engagement, all aimed at promoting ethical standards and integrating moral principles into the bedrock of care. Skills, Knowledge, and Competence represent the practical scaffold necessary for executing duties with expertise, where personal capability and dedication to professional growth, underscore conscientious care delivery. Cultural and Spiritual Influences highlight the diverse backgrounds that inform individual values and beliefs, shaping a caregiver’s approach and the nature of their professional persona. Communication and Interpersonal Dynamics focus on the relational aspects of care, emphasizing the import of a supportive environment and a deep understanding of patient needs for optimal care satisfaction. Moral Courage and Action underscore the importance of adhering to an internal ethical compass and advocating for patient rights, ensuring that care is not only proficient but also principled. Care Direction and Patient-Centric Response emphasize a targeted and responsive approach to care that aligns with patient needs and preferences, advocating for a care trajectory that is both professional and personal. Lastly, Emotional Impacts and Well-being acknowledge the spectrum of emotions intrinsic to the caregiving experience, both positive and negative, which feeds back into the professional’s emotional consequences and overall well-being. Together, these elements coalesce to define a concept rooted in ethical, empathetic, and skilled care delivery, championed by individuals who nurture their professional conduct as much as their personal growth, resonating with the moral and cultural dimensions of the communities they serve (Table 9).

**Table 9:** The result of analytic phase of development of the concept of conscience-based Nursing

Core Professional Ethics and Values: Literature Review: Manifestation of professional ethics, Ethical commitment, Moral integrity, Responsible care, Individual values, References, Positive emotions, Negative emotions Qualitative Research: Promotion of professional ethics, Institutionalization of values and beliefs, Moralism at the heart of conscientious care
Skills, Knowledge, and Competence: Literature Review: Attitude, knowledge and professional skills, Individual abilities Qualitative Research: Professional competence, Professional care as conscientious care
Cultural and Spiritual Influences: Literature Review: Arising from a religious and cultural background, Reflecting the integrity of individual values, Individual beliefs Qualitative Research: Spiritual and educational beliefs, The nature of the profession
Communication and Interpersonal Dynamics: Literature Review: Communication with the patient and teamwork companions, Supportive atmosphere Qualitative Research: Conscientious care affected by work atmosphere, Understanding patient needs, Satisfaction of patients and companions
Moral Courage and Action: Literature Review: inner voice, Moral courage, Patient rights Qualitative Research: (These themes implicitly relate to the concept of acting on one’s moral convictions, which can be linked to the idea of moral courage and the protection of patient rights.)
Care Direction and Patient-Centric Response: Literature Review: Directing care Qualitative Research: Responsive care, Professional care as conscientious care
Emotional Impacts and Well-being: Literature Review: Positive emotions, Negative emotions Qualitative Research: emotional consequences

Therefore, after this integration of theoretical and empirical insights, we arrived at the following definition of conscience-based nursing care. It can be stated as:

“Conscience-based nursing care is a balanced amalgamation of professional ethics and personal spirituality, both intrinsic and external. It reflects a nurse’s professional capability, individual belief systems, values, and the overarching sociocultural healthcare context. This approach fosters the propagation of ethical practices within a clinical setting and underscores patient-centric and responsive care. It targets well-rounded patient and familial satisfaction. However, it also acknowledges the emotional aftermaths associated with this high degree of involvement, which can swing between positive reinforcement and negative echoes for the nursing professionals”.

## Discussion

To achieve the objective of this study, which was to identify the characteristics, antecedents, and consequences of conscience-based nursing care, the results of the theoretical and work stages were analyzed and compared with other studies. Despite an extensive literature search, few studies focused specifically on the concept of conscience-based nursing care, and the definitions found in nursing texts were relatively general. However, the features identified through the integration of the theoretical and work stages demonstrate that conscience-based nursing care is a central and important concept in the nursing profession, serving as a cornerstone of ethical care and guiding nurses in their performance toward ethical practice.

A unique insight from our research is that ‘conscience-based nursing care’ interweaves professional commitment, personal experiences, academic rigour, and patient satisfaction. The diversity and richness of these attributes offer a fascinating commentary on the complexity and depth of the concept. By comparing empirical data against theoretical underpinnings, we are confronted with a multitude of layers within ‘conscience-based nursing care’. This combination of theory and fieldwork unearths aspects like the role of conscience in professional care, moralism in conscience-centric approach, and endo-exogenous spirituality in nursing.

The final definition of ‘conscience-based nursing care’ captures the holistic essence of nursing. It puts emphasis on professional abilities, personal values and beliefs, diversity in spirituality, healthcare’s social context, the promotion of professional ethics, and a commitment towards achieving patient and familial satisfaction. What is inherently unique here is the acknowledgement of nurses’ emotional consequences, ranging from positive reinforcement to emotional fatigue, a rarely discussed but potent reality of the profession. In essence, conscience-based nursing care is a multidimensional construct, balancing professional and personal, clinical, and emotional aspects. It underlines the profound impact nursing has on patients’ experiences and, in turn, how these experiences impact nurses themselves.

Our study suggests that there is potential here for further investigations. For instance, in-depth exploration of how nurses manage the emotional fallout inherent in their professional roles could pave the way for targeted interventions. Other directions might include developing strategies to maximize the positive aspects of a conscience-based approach and mitigate negative impacts.

One of the common features of the concept of conscience-based nursing care identified in both the theoretical and work stages was the importance of ethics in care. This included performing care based on courage and taking into account moral values in clinical practice.

In Jensen, Lidell [16] study, nursing colleagues described conscience as a driving force that gives them the strength and courage to have difficult discussions based on their knowledge and experience. Even when they make mistakes, they accept their shortcomings and work to correct them to maintain a clear conscience.

The literature review revealed that courage in care refers to a nurse's willingness to stand by the patient, defend their rights and needs, and take risks when necessary. It serves as a bridge between personal and professional values, enabling nurses to defend different values from the patient's perspective. Weiskopf [78] study on courage in care highlights the moral obligation of caring for prisoners, which involves respecting and maintaining their human dignity and standing up against any form of humiliation or encroachment on their dignity.

The participants in the current study also emphasized the importance of moral courage in care, which involves bearing potential threats for the benefit of the patient, maintaining a strong commitment to ethical principles, being honest when errors occur, and protecting the patient's rights. These characteristics are considered part of conscience-based nursing care, which is guided by moral courage and moralism in care. This approach leads to care that prioritizes the patient's well-being and can enhance nurses' ability to adhere to values and perform their duties with bravery.

Another feature of conscience-based nursing care mentioned both in the literature review and the field stage is the provision of professional and humanistic care. The nurses in the present study emphasized that they consider their care to be based on conscience when they can provide standard care using up-to-date knowledge at the bedside, fully utilize their professional skills during clinical practice, and remain committed to the process and professional principles of nursing continuously. Additionally, the participants emphasized the importance of non-discrimination and paying attention to adverse conditions of patients in care as characteristics of conscience-based nursing care.

Mazaheri et al. [52] noted that from the perspective of nurses, conscientiousness in clinical practice involves performing daily tasks well, being generous in helping others, going beyond the job and profession requirements, and treating others as one would like to be treated. Jasemi et al. [15] identified self-empowerment to perform the clinical role, effective time management during care provision, development of practical skills, increasing knowledge of care, and efforts to provide care beyond routine as strategies for providing conscience-based care. Therefore, it can be concluded that conscience-based performance involves professional performance aligned with the standards of care, which in some cases may require going above and beyond one's duty, while maintaining a client-centered approach.

The presence of a positive working environment is crucial for enabling nurses to perform professional care and enhancing their decision-making power. The characteristics of hospital management, nurses, and the level of support provided to nurses to deliver care all have an impact on their performance. The participants in the study noted that the level of pressure on nurses is related to their ability to share efforts in work relationships. Developing a supportive atmosphere and creating an environment of respect, trust, and open communication in the workplace has been found to be effective in reducing psychological pressure on nurses [79, 80]. For nurses to deliver conscientious performance in the healthcare system, they must be sufficiently prepared and supported [20].

In addition to working in a non-judgmental environment, nurses need legal and ethical support from nursing managers and colleagues. The experience of nurses in Jensen, Lidell [16] study demonstrated that in order to deliver conscientious care, nurses seek support and attempt to delegate their responsibilities when they become too overwhelmed with professional and ethical obligations. This can help alleviate their conscience and free them from moral obligations.

Erickson and Strandberg [29] suggest that nurses should develop their skills and knowledge in dealing with ethical issues, critical self-awareness, and effective interpersonal communication strategies through theoretical and practical training to address conscientious issues at the bedside. If nurses can share their troubled conscience with others, they can find ways to constructively deal with the issue. Revising the methods of dealing with conscientious issues presents an opportunity for nurses to come to an agreement with themselves, ultimately leading to improved performance [15].

Novice nurses working in clinical environments require additional support to deliver conscience-based care. These newly hired staff members have just entered a clinical environment with full responsibilities that are very different from the academic environment. Therefore, they need extensive support from their colleagues and managers. Providing adequate support for novice nurses reduces their stress and provides a platform for delivering conscientious and ethical nursing care. The statements of nurses in Jasemi et al. [15] study demonstrated that high expectations increase the level of individual stress and ultimately affect the quality of care provided. They also noted that the support they receive at the beginning of their work helps them manage stress and time, and provides a foundation for conscientious nursing practice [59].

Conscience is developed through our lived experiences throughout life and is an integral part of our daily and professional lives. It is not something that we can get rid of or escape from. Conscience is formed during childhood alongside family upbringing and is subject to growth throughout life. Parents play a significant role in teaching their children about conscience, and this knowledge is passed down through generations. Additionally, studies have shown that people's conscience is shaped by the social culture that governs their living environment, which is also influenced by their religious beliefs. Conscience plays a crucial role in meeting the needs of patients and ensuring high-quality care. When nurses have a strong conscience, they are empowered to deliver care based on conscience. Their spiritual beliefs during care not only do not cause torment of conscience, but also calm their conscience and increase the pleasure of care [15].

The participants in the present study also recognized that their conscience-based performance in clinical care is derived from their family upbringing and parents. They also acknowledged that their religious and spiritual beliefs guide them to deliver care based on conscience and prevent them from doing work that goes against their conscience. Based on these findings, it can be concluded that the formation of conscience in the context of exogenous and endogenous spirituality originates from an individual's spiritual and educational beliefs, and cultural background. It can ultimately lead to the delivery of conscience-based care.

Care centered on professional commitment is one of the features found in the work phase in the field, which was not present in the theoretical phase. Participating nurses considered the knowledge of the profession and the motivation to serve as a part of providing care based on conscience, and in their interviews, they pointed out that if people have a correct and sufficient knowledge and insight about the nursing profession, its duties and responsibilities, and its rights and benefits, they have more conscientious function. People who do not know about the profession when they are faced with the professional responsibilities of nursing do not accept it and somehow do not involve their conscience in the care. Considering that the success and progress of a profession depends to a large extent on the existence of a positive attitude towards it, therefore, a favorable attitude and knowledge of various aspects of that profession creates positive feelings and emotions. Therefore, it is expected that by creating proper professional self-knowledge in people, it will also affect the care performance of people.

We have sought to link the literature review to the antecedents, attributes, and consequences identified during both the theoretical exploration and the empirical field research. An essential dimension of this interconnection is the cultural context in which the research was conducted, specifically within an environment replete with Islamic traditions.

The influence of Islamic cultural values on the study findings and participants cannot be understated in the realm of conscience-based nursing care. Islamic values permeate the ethos of the caregiving environment for the nurses interviewed. For instance, the principle of 'Ihsan,' often translated to 'doing what is beautiful,' resonates with the Islamic approach to nursing. This inspires a level of care that goes beyond mere duty and enters the realm of spiritual and moral obligation. The nurses operating within this cultural milieu may naturally integrate these values into their practice, potentially affecting their interactions with patients and their overall approach to providing conscience-based care. The findings reflect how Islamic cultural values shaped the perspectives and experiences related to conscience-based nursing care among the study participants.

## Conclusion

The findings of the present study demonstrate that the use of conscience in nursing care is the foundation of ethical care that elevates clinical practice to professional care. Delivering this type of care reflects the integration of individual and social values that arise from an individual's beliefs and cultural background. This integration occurs based on professional competence, available resources, and a supportive atmosphere within the healthcare organization, which ultimately leads to the delivery of responsive care, moral integrity, individual excellence, and the development of professional nursing.

### Supplementary Information

Supplementary Material 1.

## Data Availability

No datasets were generated or analysed during the current study.
