# Supplementary material for: Concept analysis of conscience-based nursing care: a hybrid approach of Schwartz-Barcott and Kim’s hybrid model
Source: BMC Med Ethics. 2024 Jun 18;25:70. doi: 10.1186/s12910-024-01070-8 (PMC11184846; doi:10.1186/s12910-024-01070-8)
Supplement: Supplementary file 1 — Supplementary Material 1. [file 12910_2024_1070_MOESM1_ESM.docx]

The following interview guide was used for the semi-structured interviews.

Can you walk me through a typical day for you in patient care?

Can you share an experience where you had to make a difficult professional decision in a challenging situation?

Could you provide an example of a time when you provided care based on conscience?

What specific characteristics of that example led you to believe it was based on conscience?

What motivates you to practice conscientious care?

What factors contribute to you providing unconscionable care?

The interview also explored the various aspects of conscience-based care, including its defining features, causes, and consequences. To ensure that participants have shared all of their experiences, exploratory questions such as "Can you elaborate on that?" and "What do you mean by that?" were used. Finally, the interview concluded with the question, "Is there anything else you would like to add?" to give participants the opportunity to express any additional thoughts or experiences.
